# Firework-Related Ocular Trauma at a Level 1 Trauma Center During a City-Wide Pandemic Lockdown: A Case Series

**DOI:** 10.7759/cureus.48531

**Published:** 2023-11-08

**Authors:** Marycon Jiro, Georgia Kaidonis, Joey Chiang, Jay Stewart, Sriranjani Padmanabhan

**Affiliations:** 1 School of Medicine, University of California San Francisco, San Francisco, USA; 2 Department of Ophthalmology, West Coast Retina Medical Group, San Francisco, USA; 3 Department of Medicine, University of Washington, Seattle, USA; 4 Department of Ophthalmology, University of California San Francisco, San Francisco, USA

**Keywords:** open globe rupture, open globe injury, penetrating eye injury, ocular blast injury, fireworks

## Abstract

Purpose

To report six ocular injury cases caused by unlicensed fireworks and subsequent complications at a level 1 trauma center in the setting of coronavirus disease 2019 (COVID-19)-related shelter-in-place orders.

Observations

All six cases occurred between March 2020 and July 2020 and involved fireworks of non-official use. A majority of subjects were male between the ages of 17 and 53 years old. Ocular trauma presented as the following: Case 1 is a 17-year-old male who sustained a left-sided corneal abrasion and small intraocular foreign body after a firework exploded in his hand. Case 2 is a 47-year-old male who presented with a right globe rupture after being struck with a projectile from a neighborhood fireworks display. Case 3 is a 36-year-old male with corneal abrasion, traumatic iritis, and commotio retinae after a firework injury in the setting of alcohol use. Case 4 is a 35-year-old male who presented with left lid injury, corneal abrasion, and hyphema after being struck by a firework with evidence of penetrating eye trauma on subsequent exams. Case 5 is a 53-year-old male who developed bilateral subconjunctival hemorrhages and a partial-thickness corneal laceration after a firework exploded in his left hand. Case 6 is a 48-year-old woman who sustained bilateral corneal stromal foreign bodies while cooking after a firework exploded near her vicinity.

Conclusions and importance

Fireworks are a preventable cause of mortality and long-term ocular morbidity. The index of suspicion for open globe injuries related to fireworks should be high given the mechanism of injury. These presenting cases at a level 1 trauma center and safety net hospital may be an unforeseen by-product of COVID-19 lockdowns. Our findings are relevant to trauma centers and safety net hospitals with large cases of firework injuries. Further initiatives to improve awareness of the dangers of fireworks should be prioritized to limit harms for all community members.

## Introduction

Fireworks are a preventable cause of mortality and morbidity associated with significant ocular injury, frequently resulting in complete vision or visual acuity loss [1-5]. According to the United States Consumer Product Safety Commission, the majority of firework-related injuries in the United States occur annually between June 21 and July 21, corresponding to July 4th celebrations. Additionally, the increase of firework-related, emergency department (ED)-treated injuries from 2019 (10,000) to 2020 (15,600) was statistically significant, which may have been due to coronavirus disease 2019 (COVID-19) shelter-in-place mandates and cancellations of city-organized July 4th fireworks festivities [6]. We report six cases of firework-related injuries and resulting consequences at a level 1 trauma center between March 21, 2020, and July 21, 2020.

## Materials and methods

A retrospective case series of patients at the Zuckerberg San Francisco General Hospital and Trauma Center was undertaken between March 21, 2020, and July 21, 2020. Inclusion criteria included patients of all ages who presented with ocular trauma after igniting a firework or being near the vicinity of a fireworks display. Exclusion criteria were patients without ocular trauma and patients with ocular trauma without confirmed injury due to fireworks.

In total, six patients who presented with ocular injuries caused by fireworks met criteria. Demographic data, mechanism of injury, best corrected visual acuity (BCVA) at presentation, presence of penetrating eye injury, ocular injuries sustained, and other bodily injuries were tabulated and discussed. The diagnosis of ocular trauma was made through clinical ophthalmic evaluation with the aid of ophthalmic ultrasound and computed tomography (CT) of the orbit, with further expert evaluation from an experienced ophthalmologist.

## Results

Case 1

A previously healthy 17-year-old male presented to the ED after a firework exploded in his right hand, causing significant injury to the abdomen, airway, face, and right hand, including traumatic amputation of three digits. Upon admission, BCVA was 20/20 in the right eye and 20/25 in the left. Intraocular pressure (IOP) was 16 in the right eye and 13 in the left eye. On examination, he was found to have left-sided reactive ptosis without tarsal involvement and a small non-penetrating left corneal foreign body without evidence of a full-thickness corneal defect. Dilated fundus examination of both eyes was unremarkable.

The patient was treated with polymyxin B/trimethoprim eye drops. Foreign body removal was planned for the following day. Repeat exam was unchanged with the exception that BCVA-left BCVA was 20/40 -2 and improved to 20/25 -2 with repeat testing. Given the patient's excellent visual acuity and a small foreign body, the risks of removal outweighed the benefits. No further intervention was pursued.

Case 2

**Figure 1:**
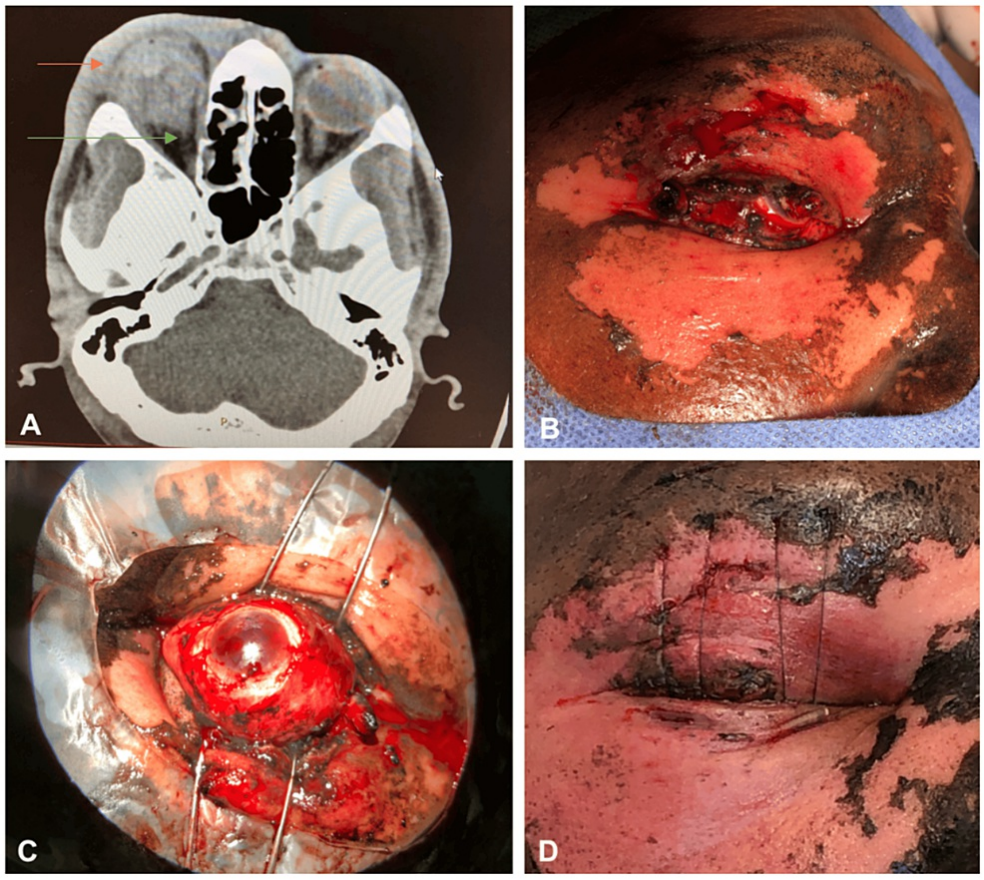
A 47-year-old male sustained an open globe injury after being struck by a firework (case 2). A: CT scan (sagittal view) showing rupture of the right globe posteriorly (green arrow) and vitreous hemorrhage (orange arrow). No intraorbital foreign body was identified. B: External photograph of the right eye. Right upper and lower lid burn injury and lacerations can be seen as well as second-degree burns to the right side of the face. C: Intraoperative external photograph of the right eye. There is diffuse subconjunctival hemorrhage, the cornea is hazy consistent with corneal burn injury, and hyphema is present. D: External photograph of the right lids following surgical repair and reconstruction. Modified Frost sutures were placed to achieve full corneal coverage and minimize cicatricial ectropion CT: computed tomography

A previously healthy 47-year-old man presented with acute vision loss after a fireworks display ignited by his neighbor struck his right eye. BCVA was 20/40 in the left eye with an IOP of 8; BCVA and IOP of the right eye could not be assessed due to lid edema. He sustained extensive injuries to the right eye including lid lacerations, severe burns to the upper and lower lids, cornea, and conjunctiva, and hyphema (Figure 1). Non-contrast CT scan of the orbits revealed a deformed right globe suggestive of globe rupture and multiple posterior chamber hyperdensities suspicious for vitreous hemorrhage (Figure 1). No orbital foreign body was identified on imaging.

The patient underwent surgical exploration of the ruptured right globe; however, the posterior globe rupture was deemed unrepairable due to the severity and location of the laceration. The upper and lower lid burn injuries involved the anterior lamellar and tarsus. The lids were debrided and lacerations were repaired by the oculoplastic service. A modified Frost suture was used to position the lids for optimal postoperative healing (Figure 1). The patient was prescribed erythromycin ointment postoperatively with 24 hours of vancomycin/ciprofloxacin. Repeat exam on postoperative day 5 was unremarkable. The patient then transferred their care elsewhere and was no longer seen at our center.

Case 3

A previously healthy 36-year-old man presented with a firework blast injury to his bilateral hands, face, and eye after a firework exploded more quickly than expected in the setting of alcohol intoxication. BCVA was 20/20 in the right eye and 20/25 in the left. IOP was 20 in the right eye and 14 in the left eye. Slit lamp examination of the right eye revealed corneal abrasion. The left eye was found to have a temporal abrasion of the bulbar conjunctiva and traumatic iritis. Dilated fundus examination was unremarkable on the right but revealed a small area of commotio retinae in the peripheral retina on the left. There was no evidence of penetrating eye injury. The patient was treated with polymyxin B/trimethoprim in both eyes without steroids. Repeat exam five days later demonstrated traumatic iritis with 1-2+ anterior chamber cell and resolution of the superficial bulbar conjunctival laceration. The patient's antibiotics were discontinued, and he was treated with prednisolone acetate 3-2-1 taper with use of artificial tears. Final BCVA was 20/20 -1 in both eyes.

Case 4

**Figure 2:**
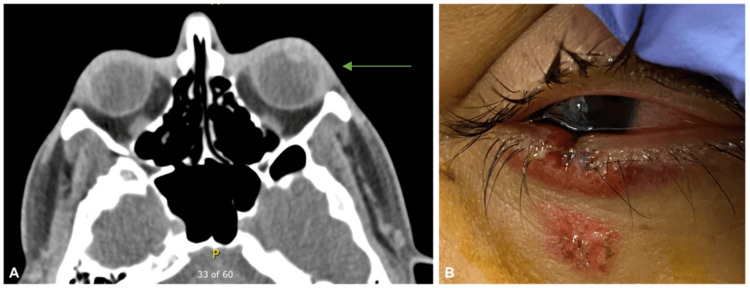
A 35-year-old male (case 4) was struck in the left eye by a projectile from a nearby firework explosion. A: CT scan (sagittal view) showing formed left globe, without evidence of intraocular or intraorbital foreign body (green arrow). B: External photograph of the left eye. There are left lower lid defect and hyphema CT: computed tomography

A previously healthy 35-year-old man presented to the ED with blurred vision one hour after a projectile from a nearby fireworks display struck his left eye (Figure 2). BCVA was 20/20 in the right eye and light perception in the left eye. IOP was 16 in the right eye and 13 in the left. Examination of the left eye revealed a lower lid margin defect, small inferior corneal epithelial defect, inferior bulbar conjunctival abrasion with adjacent subconjunctival hemorrhage (SCH), and a hyphema with no view to the iris, lens, or fundus. Seidel testing was negative. Brightness scan (B-scan) and non-contrast CT scans were performed, demonstrating a formed globe with no signs of penetrating eye injury or foreign body.

**Figure 3:**
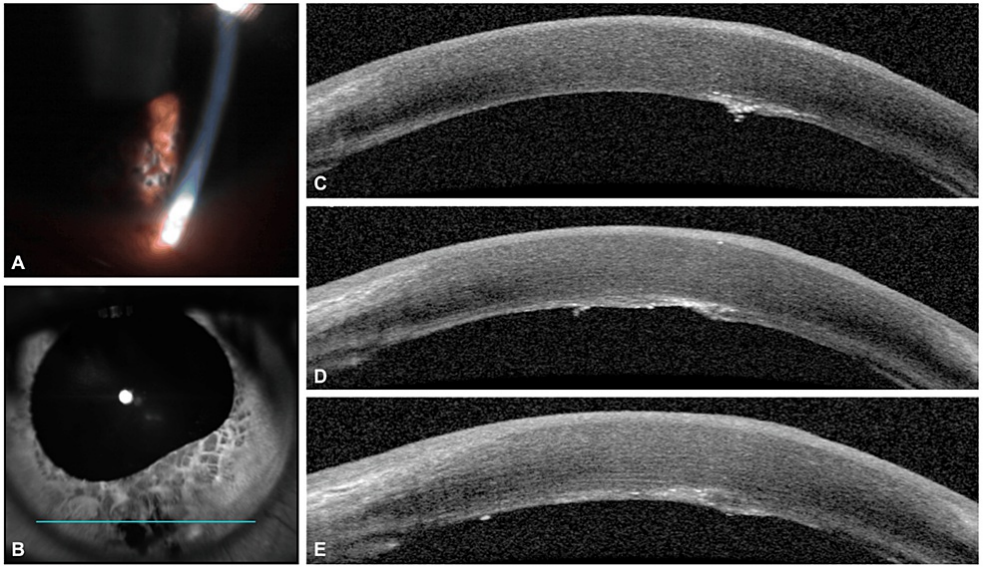
Full-thickness laceration of the cornea and iris (case 4). A: Slit lamp photograph of the left eye. There is an inferior iris defect. B-E: Anterior segment optical coherence tomography images of the left eye. En face image of the iris shows an inferior iris defect and irregular pupillary margin (B). Disruption of Descemet's membrane can be seen on cross-sectional images through the cornea (C-E) at the level of the superior aspect of the iris defect (as indicated by the blue line in B)

The patient was treated with atropine 1% one drop twice daily, prednisolone acetate 1% one drop four times daily, polymyxin B/trimethoprim one drop four times daily, and head of bed elevation. Upon follow-up five days later, BCVA improved to 20/40 on the left. Epithelial defects had resolved, and the hyphema improved. There were persistent corneal edema with focal endothelial pigment and Descemet's membrane disruption peripherally at six o'clock. The iris was irregular in the inferotemporal quadrant. Twelve days post injury, BCVA on the left had improved to 20/25 and IOP remained normal. The hyphema resolved and the inferior iris was now visible. A full-thickness defect in the inferior iris was identified in the same region as the overlying corneal injury and lid defect (Figure 3). Gonioscopy revealed a small area of cyclodialysis inferiorly, blood in the inferior angle, and inferior synechiae with no clear area of angle recession. No disruption to the lens was visualized. Dilated fundus examination revealed a small amount of vitreous hemorrhage inferiorly, without injury to the retina. No foreign body was identified. Repeat B-scan and ultrasound biomicroscopy (UBM) could not identify an intraocular foreign body.

The concern for penetrating eye injury was discussed with the patient. Given that no foreign body could be identified and the patient recovered good vision, a decision was made to continue with conservative management and observation.

Case 5

A 53-year-old man with chronic obstructive pulmonary disease (COPD), sleep apnea, and hypertension presented to the ED after a firework exploded in his left hand while he was taking it away from his son. The patient presented with BCVA of 20/25 in the right eye and 20/30 in the left. IOP was normal, 14 in the right eye and 11 in the left. Slit lamp examination was positive for focal areas of SCH bilaterally and a right corneal abrasion. Dilated fundus examination was unremarkable. The patient was treated with erythromycin ointment every four hours in both eyes and polymyxin B/trimethoprim in the right eye. Repeat cornea check the next day demonstrated improvement of the small inferior SCH and corneal laceration, with near-complete resolution of the right corneal epithelial injury. Final BCVA was unchanged. There was no concern for penetrating eye injury.

Case 6

A 48-year-old woman with unknown prior medical history was brought in by ambulance after fireworks exploded near her vicinity while she was cooking in her kitchen, causing multiple left digit amputations, bilateral tympanic membrane rupture, abrasions to the face, and bilateral eye injuries. Upon initial ophthalmology exam, BCVA was 20/30 in the right eye and 20/40 in the left eye. IOP was 17 mm Hg in the right eye and 16 mm Hg in the left eye. Slit lamp examination demonstrated bilateral lid edema and erythema, conjunctival injection, and multiple small corneal foreign bodies embedded in the epithelium and anterior stroma. Dilated fundus examination was unremarkable. There was no evidence of open globe injury.

The patient was prescribed polymyxin B/trimethoprim ointment and evaluated the next day. Examination of the right eye demonstrated a small area of pigmented superficial nasal cornea without foreign body and a completely healed epithelium. The left eye was unchanged. The remaining superficial foreign body in the left eye was removed without complications.

## Discussion

The range of eye injuries seen in our center (a level 1 trauma center that provides care at every level of injury from prevention through rehabilitation) between March 21, 2020, and July 21, 2020, is similar to that reported previously [6]. One half of cases had bilateral eye injuries, and a majority of the injuries occurred in the neighborhood near their home, a characteristic of COVID-19 pandemic injuries reported in other studies [7,8]. Corneal abrasions were found in three of six cases, open globe injuries in two of six cases, and penetrating eye injuries in two of six cases (Table 1). Two cases resulted in digit amputations, and one case involved injuries in the abdomen and legs. Similar to prior reports of firework injuries [6], the parts of the body most often injured in our patients were hands and fingers, eyes, head, and face, with only one case as a solely orbital injury. A majority of patients were male (five out of six), similar to firework-related injury studies [1,5,9]. However, our patients were often older than patients that presented with firework-related ocular injury in other studies, with a median age of 39.3 years old, and a majority of our patients aged between 35 and 55 years of age as opposed to under 18.

**Table 1:** Firework-related ocular injuries presented to the emergency department of Zuckerberg San Francisco General Hospital and Trauma Center between June 2020 and July 2020 BCVA: best corrected visual acuity; NLP: no light perception; FB: foreign body; SCH: subconjunctival hemorrhage; LP: light perception; WNL: within normal limit; OD: oculus dexter, right eye; OS: oculus sinister, left eye; OU: oculus uterque, both eyes

Case	Age (years), sex	Mechanism of injury	BCVA at presentation (OD, OS)	Penetrating eye injury?	Ocular injuries sustained	Other injuries
1	17, male	Firework explosion in the right hand	20/20, 20/25	No	OD: WNL; OS: corneal abrasion, fine mid-stromal FB	Amputated right one to three digits, abdominal and leg wounds
2	47, male	Neighborhood fireworks display struck the right eye	NLP, >20/40	Yes	OD: lid laceration and burn, corneal and conjunctival burn, hyphema, open globe; OS: WNL	Second-degree facial burns
3	36, male	Premature fireworks exploded in his hands	20/20, 20/25	No	OD: corneal abrasion; OS: conjunctival abrasion, traumatic iritis, commotio retinae	Degloving/burn injuries to hands
4	35, male	Projectile from neighborhood fireworks display struck the left eye	20/20, LP	Yes	OD: WNL; OS: lid margin laceration, hyphema, open globe	None
5	53, male	Firework exploded in his left hand as he took it away from his child	20/25, 20/30	No	OD: SCH, partial-thickness corneal laceration; OS: SCH	Degloving injury to the left hand and digits requiring surgical repair
6	48, female	Firework exploded in the left hand while cooking	20/30, 20/40	No	OU: scattered punctate corneal FBs in superficial stroma	Multiple digit amputations, tympanic membrane rupture

Of the six cases, two required a surgical or procedural intervention, while the rest were managed conservatively (Table 1). Case 2 illustrates the severity of ocular trauma that can occur from high-temperature and high-pressure conditions caused by a firework explosion. The anterior surfaces of the eye and face sustained burn injuries, while the globe ruptured posteriorly; this is consistent with a blunt trauma injury rather than a penetrating injury from a projectile. Case 4 represents an unusual scenario where a self-sealing penetrating eye injury was highly suspected despite no evidence of intraocular or orbital foreign body on clinical exam, gonioscopy, CT imaging, B-scan, UBM, and anterior segment optical coherence tomography (OCT). As no particles were recovered, the internal changes may have been due to shock wave effects from external injury. Nevertheless, given that the globe was intact and much of the anterior segment structures were initially obscured by hyphema, the extent of injury was not appreciated until subsequent exams. This highlights the need for a high index of suspicion for penetrating eye trauma following firework explosions due to the mechanism of injury.

Firework-related ocular injuries increased during the COVID-19 pandemic [10]. This increased incidence may be an inadvertent outcome of lockdowns, with prior media reports suggesting heightened firework use in several US states such as California [11,12]. Due to social distancing protocols forcing closures of celebratory events, more individuals of different age ranges may have opted to ignite unlicensed fireworks (legally or illegally acquired) in public spaces and in more enclosed spaces, increasing the risk of firework injuries for participants and bystanders which aligns with studies that show almost 99% of all ocular injuries from fireworks are due to consumer-grade and homemade fireworks [1]. Eye care professionals at our safety net hospital observed a significant increase in firework-related injuries during the COVID-19 lockdown when compared to prior years. In the event of future lockdown situations or canceled fireworks displays, clinicians may need to be aware of the increased use of unregulated fireworks and subsequent firework-related injuries. Future studies are needed to demonstrate this associated link.

The broader literature has identified a strong association between socioeconomic status and ocular trauma, especially in the construction workplace or agricultural industry [9,13-15]. Living in socioeconomically deprived areas, lower educational attainment, lower annual income, and lack of insurance correlate with higher rates of eye injuries. Furthermore, immigrants and minorities are found to have a higher incidence of reported globe injuries, which may be attributed to factors such as a lack of patient education and patient anxiety [16]. These findings are particularly relevant to trauma centers and hospitals that serve a safety net role in a marginalized community. Our center is a safety net hospital serving a largely low-income, unhoused, and diverse patient population and may care for a disproportionate amount of patients with firework injuries compared to other community hospitals. 

Limitations of our series include the lack of long-term follow-up and a small sample size, which precludes subanalysis and limits generalizability. As this series focuses on firework-related injury cases treated at a specific trauma center, this may introduce a selection bias. However, the varied cases and management may be useful for providers at other trauma centers. More research is required to further investigate the long-term impact of firework-related injuries, to improve public awareness of the risks of mishandling fireworks, and to identify and disseminate techniques for safe fireworks handling.

## Conclusions

Further initiatives to regulate firework activities and improve community awareness of the dangers of fireworks should be prioritized, especially during the ongoing pandemic, in order to limit harms associated with fireworks for all members of the community.
